# What a SMART Pharmacist Can Discover in Only One Day: A Public Health Initiative Focused on Diabetes in North Macedonia

**DOI:** 10.3390/healthcare13172107

**Published:** 2025-08-25

**Authors:** Vesna Stavrova, Maja Simonoska Crcarevska, Zorica Naumovska, Biljana Bozhinovska, Joana Andonoska, Katarina Stavric, Viktorija Maksimova, Arijana Meštrović, Michael John Rouse

**Affiliations:** 1 Pharmaceutical Chamber of North Macedonia, 1000 Skopje, North Macedonia; 2 Faculty of Medical Sciences, Goce Delcev University, 2000 Stip, North Macedonia; 3 Faculty of Pharmacy, Ss. Cyril & Methodius University in Skopje, 1000 Skopje, North Macedonia; msimonoska@ff.ukim.edu.mk (M.S.C.); zose@ff.ukim.edu.mk (Z.N.); 4 The Association of Private Community Pharmacies of the Republic of North Macedonia, 1000 Skopje, North Macedonia; bile@annifarm.com.mk (B.B.); joana.andonoska@hotmail.com (J.A.); 5 Faculty of Medicine, Ss. Cyril & Methodius University in Skopje, 1000 Skopje, North Macedonia; kstavric@hotmail.com; 6 Pharma Expert d.o.o., 10 040 Zagreb, Croatia; 7 American Pharmacists Association, Washington, DC 20037, USA; mrouse@aphanet.org

**Keywords:** pharmaceutical care, diabetes, patient safety, prevention, early detection, education, SMART pharmacists

## Abstract

**Background/Objectives:** Pharmacists’ roles are shifting from dispensing medications to managing chronic diseases and prevention. Diabetes is a growing public health issue requiring early detection and management, where pharmacists can play a key role. The SMART Pharmacist Program promotes continuing education and expanded care, and a nationwide blood sugar screening campaign in North Macedonia was conducted to evaluate the impact of pharmacists in detecting undiagnosed diabetes and supporting glycemic control. **Methods:** This descriptive observational cross-sectional study was conducted mainly on 14 November 2024, in 98 community pharmacies across 14 cities. Participants over 18 years old were recruited via voluntary sampling. A total of 998 measurements were performed on the campaign day, with additional screening extending to 24 January 2025, totaling 1085 participants. Blood glucose was measured by finger prick testing and classified according to national and NICE guidelines. A structured questionnaire collected demographic, medical, and lifestyle data. **Results:** Among 1085 participants (65.1% female, mean age 57.6 ± 14.5 years), 258 (23.8%) had diagnosed diabetes, mostly Type 2 (226; 20.8%), while 827 (76.2%) were undiagnosed. Males had 1.7 times higher odds of diabetes. Diabetes prevalence correlated with physical inactivity, higher BMI, smoking, and chronic diseases. Among the undiagnosed, 17.8% were prediabetic and 4.3% diabetic. Of diagnosed patients, 57% had well-controlled and 42% poorly controlled diabetes. Metformin-based therapies were most common for Type 2 diabetes. **Conclusions:** Community pharmacists can effectively support early detection of diabetes and identify patients with suboptimal glycemic control, enhancing diabetes management in the community.

## 1. Introduction

### 1.1. New Roles of Pharmacists in Chronic Disease Management

In the last few decades, the role of pharmacists in community pharmacy has progressively changed, especially in the management of chronic diseases [1]. Shifting from the preparation of custom medication and distribution of medicines to the provision of pharmaceutical care, pharmacists have become a health professional focused on improvement of patient health outcomes [2]. Recently, the European Directorate for the Quality of Medicines & HealthCare (EDQM) stated that they found significant variation in the acceptance and implementation of pharmaceutical care in Europe. Therefore, the Council of Europe adopted a resolution on the Implementation of Pharmaceutical Care that explained pharmaceutical care and demonstrated the pharmacist’s value to optimization and rationalization of medicine use, and to address morbidity and mortality associated with medicine-related problems [3].

The pharmaceutical care concept in North Macedonia has been established as part of wider reforms within the pharmaceutical sector. The Ministry of Health of the Republic of Macedonia published the Guidelines for the Principles for Good Pharmacy Practice (GPhP) in 2009, covering the requirements that should be fulfilled according to the GPhP guidelines, recommendations, and establishment of standards for GPhP [4]. According to Macedonian legislation, however, pharmacy services and pharmacists’ roles are described more as product-oriented services and less within the scope of patient care [5]. In February 2024, some very important changes were included in the Law on Health Protection. Specifically, in Amendment 89 regarding “Pharmacist integration and rights,” wording was added that a “pharmacist gives information and advice to the patients, as well as preventive and other services related to medication therapy.” This was a step forward in the Macedonian health system that encouraged pharmacists to be more actively included in preventive and therapeutic plans for patients [6].

Intending to enhance the new knowledge, experience, attitudes, and values, the pharmacy education system in North Macedonia has undergone some changes. The Faculty of Pharmacy in Skopje has included a course in Social Pharmacy and Methodology as an obligatory course and Drug Dispensing and Communication Skills as an elective course in their study program. An additional course named Pharmaceutical Care—specially dedicated to principles and practical recommendations on patient care that will enhance the pharmacy student’s awareness of their roles and responsibilities in the community pharmacy—has been introduced in the ongoing accreditation process [7]. The latest pharmacy curriculum at the Department of Pharmacy, Faculty of Medical Sciences in Stip, has already introduced a course named Social Pharmacy as an obligatory course in the last (fifth) year of study, and an elective course called Good Pharmacy Practice and Pharmaceutical Care [8]. These changes will provide students with an opportunity to continue such practice after graduation but also prepare them to easily pass the professional exam and become licensed pharmacists.

A plan and program for traineeship of healthcare professionals was given by the Pharmaceutical Chamber of North Macedonia (“Chamber”). The Chamber is the official body for administration of the professional examination of healthcare professionals with higher education in the field of pharmacy. It strongly supports the need for advancements in pharmaceutical care and pharmaceutical practice in community pharmacy, as well as in hospital and clinical pharmacy [9]. Therefore, the pharmacist’s internship is designed to include all those settings [10]. Moreover, a postgraduate health specialization program, “Community Pharmacy Practice,” has been offered to pharmacists by the Faculty of Pharmacy in Skopje to continue their education as healthcare practitioners focused on patient care [11].

Patient care comprises a process in which pharmacists collaborate with the patient primarily, but also with other health professionals to design, implement, and monitor therapeutic plans, resulting in optimized medicine use and providing specific therapeutic outcomes for the patient [12]. Diabetes is one of the most serious challenges for health systems throughout the world, with over 830 million diagnosed patients by 2022, with 90% prevalence of Type 2 diabetes [13,14]. Its prevalence has been rising more rapidly in low- and middle-income countries than in high-income countries. According to the World Health Organization (WHO), more than half of these patients did not take medication for their diabetes in 2022, and this is one of the reasons why complications and outcomes can be unpredictable [13]. In the Republic of North Macedonia, according to the 2022 Diabetes Registry published by the Institute of Public Health, the number of newly registered, newly diagnosed patients with diabetes is 7737 cases, and the total prevalence is 134,668 patients [15]. The growing prevalence of diabetes and prediabetes presents a significant public health challenge, requiring innovative and accessible screening strategies. Community pharmacists are uniquely positioned to deliver rapid, point-of-care interventions that combine screening, education, and patient counseling. Therefore, this study addresses the following research questions: “What is the current level of diabetes awareness, risk factor recognition, and glycemic control among community pharmacy visitors in the country?”; “What potential role can community pharmacists play in a single-day, public health initiative in this context?”; and “How can community pharmacists contribute to the enhancement of national strategies for diabetes prevention and management through targeted, short-duration interventions?”

Management and treatment strategies for Type 2 diabetes are described as complex, demanding persisting medical care, and include the importance of patient education and adherence, adequate healthy diet and lifestyle, and support to prevent acute or chronic complications [16]. Pharmaceutical care can be an important part of proper diabetes management if pharmacists are helping patients to improve their chances of reaching therapeutic and lifestyle goals. As the health professionals most educated in pharmacotherapy, pharmacokinetics, and pharmacodynamics, pharmacists can play a key role in the multidisciplinary healthcare team, contributing to better care for patients [17]. They can advise patients about the adequate dosing regimen, possible side effects of the medicines, and prospective plans if a patient is not responding with the expected outcome to the initial treatment [18]. Despite all the newest medications, patients with diabetes have difficulties in controlling their glycemic levels and managing their health condition. This is why improvement of pharmaceutical care and pharmacists focusing on patient care services are urgent needs.

### 1.2. SMART Pharmacist Program in the Republic of North Macedonia

The SMART Pharmacist Program, first introduced in Turkey, is based on the continuing professional development (CPD) approach to lifelong learning, with a particular emphasis on the application of learning into practice [19]. Based on the success and impact of the SMART Pharmacist Program in several other countries, the Chamber decided to implement a similar project in the Republic of North Macedonia [20,21,22]. The term “SMART Pharmacist” has been applied to pharmacists who complete appropriate structured competency-based education and training and then apply their learning in their practice to improve the quality of the services they provide and patient outcomes

The SMART Pharmacist Program began in North Macedonia in 2023 with the first Symposium on “Pharmaceutical Health Care,” when pharmacists conducted a personal and professional evaluation of their role in pharmacies. As a result of this initiative, the “Pharmacist Record” was created—a tool for structured documentation of pharmaceutical care that facilitates better communication with other healthcare professionals and contributes to more efficient medication therapy management.

In 2024, the topic of the second SMART Pharmacist Symposium was diabetes—a chronic condition with increasing prevalence. The blood sugar measurement campaign was a direct extension of this educational activity, focused on early detection and prevention, and the campaign provided evidence that pharmacists are prepared and educated to make a meaningful contribution to public health.

In 2025, the SMART Pharmacist Program continued with new topics: hypertension, dyslipidemia, and obesity, involving an increasing number of pharmacists and pharmacies. Through this activity, a clear vision to transform the role of pharmacists in North Macedonia from medication dispensers to active partners in public healthcare was created. The SMART Pharmacist Program was created with the slogan “*Learn Today—Apply Tomorrow*” to empower practitioners to implement their new knowledge, skills, attitudes, and values to directly improve patients’ outcomes, especially those patients with chronic conditions. The key objective of the SMART Pharmacist Program in North Macedonia was to elevate the role of pharmacists in preventive healthcare by enabling them to contribute to the early detection of chronic diseases such as diabetes, but also to the management of therapy for patients with diabetes. The aims were to motivate pharmacists in their learning, promote their continuing professional development based on real-world challenges in pharmacy practice, and encourage the application of the learning to improve patient outcomes.

Therefore, this study aims to discuss the previously mentioned research questions of this paper within the context of the current opportunities for pharmacists and their evolving role in enhancing the therapeutic outcomes of patients with Type 2 diabetes. Specifically, the study focuses on the proactive involvement of community pharmacists in glycemic screening initiatives, both for individuals already undergoing treatment and for the identification of previously undiagnosed cases of hyperglycemia. Such initiatives have been encouraged during WHO Diabetes Day campaigns, emphasizing pharmacists’ commitment to public health. The SMART Pharmacist Program provided the initiative to integrate community pharmacists into specific patient care activities, thereby expanding their responsibilities beyond traditional drug dispensing roles. Through this program, as shown in previous studies in Serbia, Turkey, and Montenegro, pharmacists engage in patient education, medication management, and the early detection of diabetes, contributing significantly to the overall management of the disease [19,20,21,22]. As part of the SMART Pharmacist Program for continuing education, the Association of Private Pharmacies of the Republic of North Macedonia (ZPARSM), in collaboration with Pharma Expert, an educational agency, conducted a major public health initiative for blood sugar screening in community pharmacies across the country.

### 1.3. Study Objectives

The main study objective was to identify what a SMART Pharmacist can discover in a single public health campaign, which was supposed to last only for one day. It was expected that data collection would be continued by some pharmacies to take advantage of the initiative. It was also expected that many participants would have glucose levels out of the recommended range, despite the fact that some of them were already diagnosed and receiving therapy.

## 2. Materials and Methods

### 2.1. Participants, Recruitment and Settings

The study was designed as a descriptive observational cross-sectional study with elements of screening epidemiology. The study was conducted after obtaining approval from the Ethics Committee of the Faculty of Pharmacy, Ss. Cyril and Methodius University in Skopje, North Macedonia No. 02-284/3 obtained on 29 April 2024. Additionally, given the significant impact of this study among pharmacists, to expand and deepen research activities across the country, the Faculty of Medical Sciences at Goce Delcev University in Stip, North Macedonia issued the Ethical Approval as well. 

Participants, including both patients and community pharmacists, were recruited through voluntary convenience sampling. Individuals over 18 years of age who visited participating pharmacies during the campaign and expressed interest in the activity were invited to participate, provided they gave written informed consent. Participation was entirely voluntary and free of charge. It was conducted on 14 November 2024, World Diabetes Day, across community pharmacies in the Republic of North Macedonia that voluntarily participated. The campaign was carried out in 98 pharmacies across 14 cities, with a total of 998 measurements performed in a single day. However, some pharmacies continued the activity beyond that date. The data presented here include records up to 24 January 2025, with total of 1085 entries

### 2.2. Questionnaire

The study utilized a structured questionnaire consisting of 18 questions categorized into the following sections: Demographic data, Medical history and health status, Lifestyle habits and risk factors, Details of blood glucose measurement, and Participant awareness and willingness to engage in similar future activities. The questionnaire included 12 closed-ended questions and 6 open-ended questions related to age, type of diabetes therapy, Body Mass Index (BMI), and chronic disease medications that could influence blood glucose levels. For the latter, pharmacists assessed and recorded relevant medications based on a predefined list of drugs known to impact glycemic levels including glucocorticoids such as prednisone, hydrocortisone, and methylprednisolone; antipsychotics like olanzapine, quetiapine, and risperidone; diuretics, particularly thiazide diuretics such as hydrochlorothiazide and chlorthalidone; antidepressants including maprotiline; certain antihypertensive drugs such as beta-blockers, which may affect glucose metabolism; and some HIV/AIDS medications, notably nevirapine. Adherence to therapy was assessed through a self-reported item asking participants whether their therapy was taken “Regularly” or “Not taken regularly.” Additional open-ended questions covered the measured blood glucose value and the participant’s contact information (email or phone number) for potential involvement in future public health activities.

The questionnaire was developed through iterative discussions with patients and pharmacists to ensure clarity, comprehensibility, and relevance. A pilot version was distributed to five patients and five pharmacists to assess its usability and content validity. This pilot study evaluated comprehension, acceptability (including question phrasing, complexity, and ambiguity of proposed answers), and the time required for completion. Participants were encouraged to provide feedback and suggestions for refinement. Content validity was assessed through expert review by the participating pharmacists. Insights gained from the pilot study facilitated the development of the final anonymous questionnaire, which comprised 18 refined items. Reliability analysis was conducted using Cronbach’s alpha on a subset of four items related to lifestyle (smoking, diet, physical activity, and BMI), yielding an alpha coefficient of 0.93, indicating excellent internal consistency for this domain. 

### 2.3. Training of the Community Pharmacists to Participate in the Study

Community pharmacists participating in the study received prior training as part of the SMART Pharmacist Diabetes Module 2 certified by the Chamber, conducted from 1–3 April 2024, in collaboration with Pharma Expert, ZPARSM, and the Center for Family Medicine at the Medical Faculty, Ss. Cyril and Methodius University in Skopje, Republic of North Macedonia. The training was organized as a two-day interactive workshop, covering pharmaceutical care in diabetes. Participants used training materials containing facts about available pharmacotherapy, therapeutic goals to be achieved in diabetes management, lifestyle modifications needed for patients with diabetes, and possible complications and interventions in patient care. In the practical part of the workshop, the process of foot examination, application of insulin, and methods for monitoring glucose and HbA1C were presented, as well as case studies and knowledge assessment for the participants. Learning materials and methodology were developed by Pharma Expert, an educational company, in collaboration with the local team. Materials were available in the Macedonian language to avoid misunderstandings and, additionally, fact sheets were updated and aligned with national regulations and guidelines. The trainer and author of this material was an experienced pharmacist—an assistant professor in pharmaceutical care with extensive experience in teaching and practice, and with additional training in diabetes patient care. Training also included a check list and two suggested protocols; one was for counseling patients diagnosed with diabetes and the other for early detection and screening, to achieve standardization among pharmacists delivering the service in practice.

### 2.4. Point-of-Care Testing in Community Pharmacy

Measurement of the blood glucose level was performed by finger prick testing using On Call^®^ Plus Blood Glucose Monitoring System (Acon, Tustin, CA, USA) and Medisign Mm1100 Diabetic Blood Glucose Meter (Tianjin Empecs Medical Device Co., Ltd., Tianjin, China). Data collection was carried out through a linked Google Form (Google Forms, Google LLC, https://forms.google.com), which community pharmacists completed on-site at the time of measurement.

Blood glucose levels were classified according to the Macedonian National Guideline for Evidence-based Diabetes Management and the UK National Institute for Health and Care Excellence (NICE) guidelines [23,24]. Measurements were categorized based on timing relative to food intake as fasting, 2 h postprandial, or 30 min postprandial (random). The classification thresholds for normal, prediabetes, and diabetic ranges were defined as follows:

Fasting glucose (mmol/L): Hypoglycemia < 3.9; Normal 3.9–5.6; Prediabetes 5.7–6.9; Diabetic ≥ 7.0.

2 h postprandial glucose (mmol/L): Normal ≤ 7.8; Prediabetes 7.9–11.0; Diabetic ≥ 11.1.

30 min postprandial (random) glucose (mmol/L): Diabetic ≥ 11.1.

These criteria were applied to classify participants’ blood glucose levels into normal, prediabetic, or diabetic categories.

For BMI calculation, the values were recorded as numerical entries and classified into standard BMI categories: underweight (<18.5 kg/m^2^), normal weight (18.5–24.9 kg/m^2^), pre-obese (25.0–29.9 kg/m^2^), obese class I (30.0–34.9 kg/m^2^), obese class II (35.0–39.9 kg/m^2^), and obese class III (≥40.0 kg/m^2^). These BMI categories are based on the WHO criteria [25]. The chronic disease score for each patient was calculated by assigning a value of 1 for each chronic disease listed in the questionnaire if present and 0 if absent. Consequently, the total score ranged from 0 to 10, representing the number of chronic conditions reported by each patient.

Statistical analysis was conducted using IBM SPSS Statistics 30 (trial version, IBM Corp., Armonk, NY, USA), including descriptive statistics and cross-tabulation analysis. Subsequent analyses were conducted using Python-based data analysis tools (version 3.11; Python Software Foundation, https://www.python.org/), incorporating both descriptive and inferential statistical evaluations, applied as appropriate to the dataset.

## 3. Results

### 3.1. Overview of Demographic Data of the Participants

The study was conducted in 98 community pharmacies across the Republic of North Macedonia, representing approximately 8.5% of the country’s 992 registered community pharmacies that are in the network of the National Health Fund. A total of 1085 participants were included. The respondents included both genders, with 706 females comprising 65.1% and 379 males 34.9%. The mean age of participants was 57.62 ± 14.53 years, ranging from 18 to 91 years. The average BMI calculated from 667 available numerical data was 27.5 ± 4.82, ranging from 17.2 to 56. The average blood glucose level was 6.89 mmol/L ± 2.74, ranging from 2 to 33.33 mmol/L. The detailed demographic data of the participants are presented in Table 1.

To explore how the available variables relate to diabetes status (without diabetes or diagnosed diabetes), a cross-tabulation and statistical analysis using Pearson’s chi-square (χ^2^) test were conducted (Table 2).

The analysis revealed a statistically significant association between gender and diabetes status (χ^2^ = 12.756, *p* < 0.001). Among the 1085 individuals, 144 out of 706 females (20.4%) and 114 out of 379 males (29.8%) were diagnosed with diabetes, suggesting a potential gender-based difference in prevalence. The odds ratio (OR = 1.679, 95% CI: 1.262–2.234, *p* < 0.001) indicated that males are 1.7 times more likely to have diabetes compared to females. Although this relationship was statistically significant, the effect size was small (Phi (φ) = 0.108, Cramer’s V (*V*) = 0.108).

Examining physical activity and diabetes status, a statistically significant association (χ^2^ = 10.234, *p* = 0.017) was revealed. Diabetes prevalence was highest among individuals with irregular exercise patterns (177 of 656; 27%), while those who exercised daily had a lower prevalence (37 of 182; 20.3%). Those exercising 1–2 times per week (29 of 150; 19.3%) and 3–4 times per week (15 of 97; 15.5%) showed the lowest prevalence. Effect size calculations (φ = 0.097, *V* = 0.097) indicated a weak association, with exercise frequency slightly improving diabetes prediction (Uncertainty Coefficient = 0.006, *p* = 0.004).

The analysis of BMI and diabetes status revealed a statistically significant association (χ^2^ = 21.171, *p* = 0.002). Diabetes prevalence was highest among individuals in the obese class III category (3 of 7; 42.9%), followed by obese class I (52 of 156; 33.3%), pre-obese (77 of 289; 26.6%), and obese class II (10 of 39; 25.6%). In contrast, the underweight category had the lowest prevalence (2 of 10; 20.0%), though the sample sizes for this group (10) and the obese class III group (7) were small. The effect size (φ = 0.139, *V* = 0.139) suggested a moderate association, with BMI category moderately improving diabetes prediction (Uncertainty Coefficient = 0.018, *p* = 0.002).

Further analysis showed a statistically significant association between smoking status and diabetes status (χ^2^ = 14.234, *p* = 0.003). The cross-tabulation revealed varying diabetes prevalence across smoking groups: 31.4% (37 of 118) of ex-smokers, 12.3% (9 of 73) of individuals with missing smoking data, 26.1% (153 of 587) of non-smokers, and 19.2% (59 of 307) of current smokers had diabetes. Interestingly, non-smokers exhibited the highest proportion of diabetes, while current smokers had the lowest, contrary to typical expectations. The effect size (φ = 0.115, *V* = 0.115) suggested a weak relationship, with smoking status contributing minimally to diabetes prediction (Goodman and Kruskal’s tau = 0.013, *p* = 0.003). These results may be influenced by confounding factors such as age, lifestyle, or pre-existing conditions, warranting further investigation.

The relationship between diabetes status and chronic disease revealed a strong association (χ^2^ = 79.499, *p* < 0.001). Diabetes prevalence was higher among individuals with other chronic diseases (222 of 684; 32.5%) compared to those without chronic disease (35 of 401; 8.7%). Effect size calculations (φ = 0.271, *V* = 0.271) indicated a moderate significant association, with diabetes status significantly improving the prediction of chronic disease (Uncertainty Coefficient = 0.068, *p* < 0.001). The odds ratio analysis indicated that individuals with diabetes were approximately five times more likely to have a chronic disease (OR = 5.058, 95% CI: 3.453–7.341).

The relationship between diet and diabetes status showed no statistically significant association (χ^2^ = 7.099, *p* = 0.312). While varying diabetes prevalence was observed across different diet groups, with the highest in the region-traditional diet group (162 of 692; 23.4%) and in the balanced diet group (81 of 310; 26.1%), other categories such as macrobiotic (1 of 3), vegan (2 of 3), and vegetarian (2 of 11) had too few cases to draw meaningful conclusions. Although the effect size (φ = 0.081, *V* = 0.081) suggested a minimal influence of diet on diabetes, its small magnitude further aligns with the lack of a meaningful association between them in the available data.

### 3.2. Subgroup Analyses

The next analysis aimed to identify patients with potential blood glucose regulation issues. Using Python for data analysis, the authors applied criteria based on national and international guidelines to assess blood glucose levels in both diabetic and non-diabetic individuals. For non-diabetic patients, glucose levels were categorized according to their relation to food intake—fasting, postprandial (at least 2 h before measurement), or random check (less than 30 min before measurement). Based on these classifications, blood glucose levels were further grouped as normal, glucose-impaired (prediabetes), or suspected diabetes (diabetic). For diabetic patients undergoing therapy, glucose control was evaluated to determine whether their glycemic management was well-controlled or poorly controlled based on therapeutic targets.

Out of 827 patients without a prior diabetes diagnosis, 826 provided information on food intake before glucose measurement. Blood glucose levels were classified according to the Macedonian National Guideline for Evidence-based Diabetes Management and the UK National Institute for Health and Care Excellence (NICE) guidelines [23,24]. The results were categorized into normal, prediabetes, and potentially diabetic groups based on the recommended target blood glucose levels (Table 3).

The analysis revealed that 147 patients (17.78%) of those without a prior diabetes diagnosis (827) were categorized as prediabetic, representing 13.55% of the total screened population (1085 patients). Additionally, 35 patients (4.32%) of the undiagnosed group were classified as diabetic, representing 3.23% of the total study population. Furthermore, 10 patients were diagnosed with hypoglycemia, including 8 cases of Level 1 (3.0–3.8 mmol/L) and 2 cases of Level 2 (<3.0 mmol/L).

Out of the 258 diabetic patients screened in the study, 226 (87.6% of the diabetic population or 20.8% of the total screened population of 1085 patients) had Type 2 diabetes. Fifteen patients (5.8% of the diabetic population or 1.4% of the total screened population) had Type 1 diabetes, while nine patients (3.5% of the diabetic population or 0.8% of the total screened population) had other types of diabetes. For eight patients who reported having diabetes, the specific type was not documented.

In the Type 1 group, there were 11 females with average age 60.45 ± 12.73 (range 25–77) and 4 males, with an average age of 66.5 ± 8.38 years (range: 57–80 years). One patient did not specify their type of therapy, while two were on insulin alone, two were on a combination of metformin and insulin, eight were on metformin only, one was on metformin and vildagliptin, and one was on metformin and repaglinide. All patients reported consistent adherence to their therapy. Although treatment with metformin alone is not consistent with clinical guidelines for Type 1 diabetes, these data are presented as reported by patients during the campaign, and possible misclassification of diabetes type or incomplete reporting of therapy cannot be excluded. Regarding glucose measurements, fasting glucose levels were recorded in six patients, ranging from 4.0 to 17.1 mmol/L. In eight patients, glucose was measured two hours postprandially, with levels ranging from 5.3 to 17 mmol/L. In one patient, glucose was measured less than 30 min postprandially, with a recorded level of 13.3 mmol/L.

In the Type 2 diabetes group, there were 105 male and 129 female patients. The average age in the male group was 64.81 ± 9.96 years, ranging from 42 to 89 years, while in the female group, the average age was 65.42 ± 9.79 years, with a range of 20 to 86 years. Only 14 patients reported inconsistencies in their therapy adherence. Among the 212 patients who reported having Type 2 diabetes and specified their therapy, three patients indicated using only oral tablet therapy, while for one patient, only the presence of therapy was noted without further details. Monotherapy was the most frequently utilized treatment approach, with metformin as the single agent in 117 of 212 patients, accounting for 55% of the total cohort. Other monotherapies were less common but included insulin, which was prescribed to five patients, repaglinide to four patients, and glibenclamide to three patients. Additionally, gliclazide, glimepiride, and empagliflozin were each prescribed as standalone therapies for one patient.

Combination therapies were also implemented, with metformin serving as the cornerstone in 74 additional regimens. The most frequently observed dual therapy was metformin combined with repaglinide, prescribed in 26 patients, followed by metformin with glibenclamide in 9 patients. Metformin in combination with gliclazide was used in 7 patients, while the combination of metformin and insulin was prescribed in 10 patients. Other dual therapy regimens included metformin with glimepiride in five patients, metformin with empagliflozin in two patients, and metformin with sitagliptin in one patient. Furthermore, a fixed-dose combination of sitagliptin and metformin hydrochloride was recorded in one patient.

Triple therapy regimens were also identified in the cohort. The combination of metformin, gliclazide, and a fixed-dose sitagliptin/metformin hydrochloride formulation was observed in two patients. A regimen consisting of metformin, gliclazide, and vildagliptin was prescribed in four patients. Furthermore, metformin, glimepiride, and insulin were used in one patient, while metformin, repaglinide, and insulin were administered in two patients.

This analysis highlights the predominance of metformin-based therapies, either as monotherapy or as part of combination treatments, reinforcing its central role in the management of Type 2 diabetes within the studied cohort.

Regarding glucose measurements, fasting glucose levels were recorded in 69 patients, with values ranging from 4.9 to 17.1 mmol/L. In 100 patients, glucose levels were measured two hours postprandially, with recorded values ranging from 4.9 to 33.33 mmol/L. Additionally, in 57 patients, glucose was measured within 30 min after a meal, with levels ranging from 5.1 to 21.6 mmol/L.

In the group with other types of diabetes, there were five males with an average age of 63.8 ± 9.36 years (range 49–74 years) and four females with an average age of 59.29 ± 9.63 years (range 44–70 years). Metformin was used by five patients, while one individual was prescribed a combination of metformin and vildagliptin. Three participants did not specify therapy for diabetes. Consistent adherence to the prescribed regimen was observed in six individuals, while two followed their regimen inconsistently, and one had unspecified adherence. Regarding blood glucose measurements, fasting levels were recorded in four individuals, ranging from 6.5 to 9.7 mmol/L. Postprandial measurements taken within 30 min after a meal were recorded in two cases (5.6 and 7.3 mmol/L), while levels taken more than two hours after a meal were observed in three individuals, with blood glucose levels ranging from 5.6 to 15.6 mmol/L.

According to Macedonian national guidelines, in this study, three patients had fasting blood glucose levels lower than the recommended 4.4 mmol/L (Table 4). A total of 147 of 258 patients (57% of the screened diabetic population) had well-controlled diabetes, while 108 of 258 patients (42% of the diabetic population) had poorly controlled or uncontrolled diabetes.

## 4. Discussion

One of the conclusions of previous studies about diabetes management in the Republic of North Macedonia was that better identification and monitoring of citizens with diabetes was needed [26]. Recent recommendations for the South-Eastern European (SEE) region from Eastern and Southern Europe Diabetes and Obesity Expert Group underline the significance of personalized and early intensive therapy, considering that the management of comorbidities can delay disease progression and diminish the risk of cardiorenal complications of diabetes [27]. Therefore, this research is important to raise awareness regarding the potential value that community pharmacists can demonstrate in the area of public health.

According to Macedonian national guidelines, well-controlled diabetes is defined by fasting blood glucose levels between 4.4 and 7.2 mmol/L, while postprandial levels should be below 10 mmol/L [26]. The study has shown that only 57% of the screened diabetic population had well-controlled diabetes, while 42% of the diabetic population had poorly controlled or uncontrolled diabetes. Due to clinical inertia in the healthcare system, those findings might have stayed undiscovered. Clinical inertia is a key obstacle that leads to suboptimal care in patients with Type 2 diabetes mellitus (T2DM). It is present in approximately 24% of those patients and it can occur at any stage of T2DM treatment [28]. Some studies show that therapeutic goals in diabetes can be achieved sooner if patients are exposed to pharmacist-led management programs in diabetes [29]. Therefore, such programs should be established not just in clinics, but also in community pharmacy settings, as this research study underlines.

One systematic review of randomized controlled trials from 2016 evaluated the effects of several pharmacist interventions carried out in various countries and in different healthcare settings, such as community pharmacies, primary care clinics, and hospitals [30]. The findings clearly support the involvement of pharmacists as members of healthcare teams in the management of patients with Type 2 diabetes, with cost-effectiveness proven in some of those studies. Not many studies are dedicated to the community pharmacy setting; studies are predominately in hospital settings or involve a special group of patients, such as those with transplants or kidney diseases [31,32,33]. Generally, studies showed improved outcomes in the patients with diabetes, including clinical, patient-reported, and medication safety outcomes [34].

In this study, the percentage of patients who had achieved the therapeutic goal in diabetes was less than 60%. These findings are aligned with research from the USA, where those numbers were just over 50% over the years. Achievement of individualized targets varied by age group and presence of comorbidities but exhibited similar trends [35]. According to previous research, the desired target with improved glycemic control is usually achieved by treatment intensification [36]. Counseling patients about lifestyle and healthy habits, like regular exercise, maintaining healthy body weight and nutrition, and avoiding smoking, are some of the most successful strategies to achieve goals in diabetes treatment [37]. Our study confirmed that all of these factors are directly associated with the diabetes status of patients who were screened in the pharmacy. The observed statistically significant association between physical activity and diabetes status in our study is consistent with the Consensus Statement of the American College of Sports Medicine, which affirms the established link between regular physical activity and improved diabetes outcomes [38]. Positive correlation in the results of this study was also found between BMI and diabetes prevalence, with an even stronger association between other chronic diseases and diabetes prevalence. This type of association was also proposed by another recently published study, indicating that BMI appears to be a substantially larger predictor of diabetes than physical activity in a large population-level sample of US adults [39].

On the contrary, our results indicated no statistically significant association between diet type and diabetes status, but these findings were also suggested by Xu et al. (2013) [40], who noted that diet type in isolation may not exert a substantial influence on diabetes prevalence, but its interaction with physical activity patterns could be critical. This reinforces the importance of evaluating lifestyle factors synergistically rather than as independent predictors in diabetes risk assessments [40].

Pharmacists in community practice can play both roles: collaborating with medical doctors if the therapy needs to be intensified, as well as advising and educating patients about healthy lifestyle. In this context, strengthening the role of pharmacists in diabetes management requires targeted policy development and regulatory support. Formal integration of pharmacists into the National Program for Diabetes could enable structured patient education, medication review, and adherence monitoring as part of multidisciplinary care teams. Amendments to the regulatory framework may allow pharmacists to perform basic clinical measurements, such as capillary blood glucose testing, along with access to patient medical records integrated into national e-health system records (MojTermin) [41].

There has been increasing reporting in the literature in the last 30 years about intervention activities provided during pharmacist-led diabetes management in pharmacy. The type of intervention has included diabetes education, medication review, drug consultation/counseling, clinical intervention, lifestyle adjustment, self-care, peer support, and behavioral intervention. Most studies used a combination of two or more categories of intervention strategy when providing services, with no specific pattern between the service model and patient outcomes [42].

Public health campaigns in many countries have shown effective results in the early detection of diabetes and prediabetes. Our results show a high percentage of prediabetes (17.78%) and 4.3% newly diagnosed diabetes, which highlights the important role of the pharmacist in diabetes screening of at-risk populations [43].

According to the latest (11th) edition of the International Diabetes Federation (IDF) Diabetes Atlas, 140,800 people aged 20 to 79 years were registered with diabetes in Macedonia in 2024, representing a 7.4% age-standardized prevalence. The data also shows that about a third of people with diabetes—33.5%—are undiagnosed, meaning that about 45,000 people in the country are living with diabetes without knowing it [44]. This finding is aligned with expectations from the literature [45,46]. The role of the pharmacist in early diagnosis of the disease is very important to highlight. It is programs such as these that may allow for earlier diagnosis and, consequently, better control of the disease with fewer long-term complications.

Joint initiatives between health professionals, such as collaborative diabetes screening campaigns between community pharmacies and general practitioners, can play a pivotal role in the prevention and diagnosis of diabetes, which may lead to a reduction in the burden to health systems and society [47,48,49,50]. Additional strategies, such as interprofessional workshops, a data-sharing platform, and communication campaigns, should be considered to spread awareness of the new roles that can be played by pharmacists in North Macedonia. Based on this study, a good strategy could be for pharmacists to participate in professional development activities like the SMART Pharmacist Program. As previous research has suggested, such strategies could also promote collaboration with general practitioners to ensure the continuity of care and follow-up of patients at high risk. For future campaigns, it should be considered that the service was relatively easy to perform and feasible in practice, but it would require more support to ensure its sustainability, and broader implementation, as confirmed in the literature [50].

Limitations of the study include the small number of participants in some demographic groups, which limited statistical analysis. There were variations in sampling the patients; thus, there are some measurements of fasting glucose, some after meals, and some between meals. In addition, the blood glucose screenings were performed on various glucometers, causing possible inconsistencies in data interpretation. It is well known that variability in glucometer readings can arise from both the device itself and the timing of measurements, leading to discrepancies in reported glucose levels. These variations can be influenced by user technique, environmental factors, and physiological conditions [51].

To avoid such scenarios, and to minimize the variability, participants in this study were instructed to use proper technique, and to record the timing of the measurement relative to food intake. They were encouraged to follow manufacturer’s instructions for blood application and meter use, as well as to store strips in a cool, dry place and to ensure meters were properly calibrated. 

Possibilities to announce this campaign publicly were limited due to the lack of regulation. Patients’ responses about lifestyle, habits, and chronic conditions were subjective and self-reported, which might have led to some patients providing “expected” answers to questions. For example, while 54 participants reported a diagnosis of obesity, BMI-based classifications indicated a higher prevalence of obesity, reflecting differences between self-reported diagnoses and objective measures. A better understanding of the complex interplay between factors such as gender, diet, physical activity, BMI index, smoking, chronic diseases, diabetes status, and blood glucose levels is needed. Each of these variables may be individually associated with diabetes, but their combined effects warrant more in-depth exploration.

Further studies should explore the implementation and impact of blood glucose measurement as a pharmaceutical service on patient outcomes. Research is also needed to identify effective strategies for enhancing pharmacist training and increasing patient engagement in diabetes management. To sustain and expand the campaign, ongoing public awareness initiatives, support from healthcare authorities, and continuing professional education for pharmacists will be essential. Building on these insights, the authors propose the formal recognition of blood glucose measurement as a pharmaceutical service in North Macedonia, with integration of results into electronic pharmaceutical records accessible to general practitioners. This approach could strengthen collaboration between pharmacists and physicians, ultimately optimizing patient-centered care and therapy management.

## 5. Conclusions

In conclusion, this study demonstrates that community pharmacists can play a valuable role in the early detection, monitoring, and ongoing management of diabetes in North Macedonia. With only one public health campaign, community pharmacists have discovered almost 300 patients with glucose levels out of range, either in undiscovered prediabetes or diabetes, or in diagnosed patients who had not achieved their therapy goals, risking complications and lower quality of life. Results demonstrate that less than 60% of screened diabetic patients had achieved targeted glycemic control, and these findings highlight a significant gap in diabetes care that might otherwise have remained undetected due to clinical inertia. The statistically significant associations observed between physical activity, BMI, comorbidities, and diabetes status reinforce the importance of integrating lifestyle assessment into the screening services of community pharmacists. In this way, pharmacies through the SMART Pharmacists Program could develop the potential to become important health centers that, through appropriate continuing professional development, application of learning into practice, and following modern protocols, position themselves as a key part of multidisciplinary patient care.

From a public health perspective, this research provides the first large-scale evidence obtained by pharmacists in a study in North Macedonia of the effectiveness of pharmacists to generate relevant clinical data and to identify patients requiring a clinical intervention. The major conclusion based on collected data was that significant impact could be achieved even if the initiative occurs in only one day; we might therefore ask how much more could be achieved if community pharmacists were motivated and empowered to provide these services every day.

By formally integrating pharmacists into the National Program for Diabetes, enabling access to the MojTermin e-system, and authorizing point-of-care testing in community pharmacies, the healthcare system could leverage pharmacists’ accessibility to strengthen early diagnosis, patient education, and therapy optimization.

For the broader healthcare system, the study’s findings support further development of targeted policies, reimbursement models, and interprofessional collaboration frameworks that would allow pharmacist-led services to operate sustainably. This approach has the potential to reduce the burden of diabetes complications, improve population health outcomes, and contribute to cost-effective, patient-centered care at a national level.

## Figures and Tables

**Table 1:** Demographic data of the participants in the study.

Category	Subcategory	Number of Respondents	% of Total Surveyed Population (1085)	Diabetes Prevalence (%)
Gender	Female	706	65.1	20.4
	Male	379	34.9	29.8
Diabetes status	Undiagnosed	827	76.22	/
	Diagnosed diabetes	258	23.78	/
Type of diabetes	Type 1	15	1.38	/
	Type 2	226	20.83	/
	Other	9	0.83	/
Smoking Status	Non-smoker	587	54.1	26.1
	Smoker	307	28.3	19.2
	Former smoker	118	10.9	31.4
Physical Activity	Not regular	656	60.5	27
	1–2 times per week	150	13.8	19.3
	3–4 times per week	97	8.9	15.5
	Daily	182	16.8	20.3
Diet	Balanced (moderate)	310	28.6	26.1
	High in sugars	47	4.3	14.9
	Vegetarian	11	1	*
	Traditional for the region	692	63.8	23.4
	Macrobiotic	3	0.3	*
	Vegan	3	0.3	*
	Other	19	1.8	*
Chronic diseases except diabetes	No chronic disease	391	36	8.7
	Chronic disease	694	64	32.5
Types of chronic disease excluding diabetes	High blood pressure	456	42.0	38.4
	Cardiovascular disease	195	18.0	39.5
	Other	132	12.2	26.5
	Obesity	54	5.0	37
	Dyslipidemia	168	15.5	10.7
	Liver disease	18	1.7	*
	Inflammatory diseases	32	2.9	*
	Polycystic ovary syndrome	6	0.6	*
	Kidney disease	6	0.6	*
	Sleep apnea	8	0.7	*
BMI categories	Underweight	10	0.9	20
	Normal	246	22.7	15
	Pre-obese	290	26.7	26.6
	Obese category I	157	14.5	33.3
	Obese category II	37	3.4	25.6
	Obese category III	7	0.65	42.9
Blood glucose measurement timing	Fasting	375	34.6	/
	Postprandial (>2 h)	467	43	/
	Postprandial (<30 min)	242	22.3	/

* Prevalence not calculated due to small sample size.

**Table 2:** Association between diabetes status and selected variables: statistical results.

Statistics (n = 1085)	Chi-Squared Value	Chi-Squared *p*-Value	Odds Ratio (95% CI)	Phi-Coefficient Value	Cramer’s V Value	Uncertainty Coefficient/Tau
Gender vs. Diabetes status	12.756	<0.001	1.679 (1.262–2.234)	0.108	0.108	/
Physical activity vs. Diabetes status	10.234	0.017	/	0.097	0.097	0.006 (*p* = 0.004)
BMI vs. Diabetes status	21.171	0.002	/	0.139	0.139	0.018 (*p* = 0.002)
Smoking status vs. Diabetes status	14.234	0.003	/	0.115	0.115	0.013 (*p* = 0.003)
Chronic disease vs. Diabetes status	79.499	<0.001	5.058 (3.453–7.341)	0.271	0.271	0.068 (*p* < 0.001)
Diet vs. Diabetes status	7.099	0.312	/	0.081	0.081	/

**Table 3:** Classification of non-diabetic patients based on blood glucose levels and measurement timing relative to food intake.

Measurement	Defined Glucose Range (mmol/L)	Number of Patients	Observed Range	Category
Fasting (295 patients)	<3.9	10	2–3.8	Hypoglycemia
	3.9–5.6	143	3.9–5.6	Normal
	5.7–6.9	114	5.7–6.9	Prediabetes
	≥7	28	7–14.3	Diabetic
2 h postprandial (351 patients)	≤7.8	312	3.1–7.8	Normal
	7.9–11	33	7.9–10.7	Prediabetes
	≥11.1	6	11.1–17.4	Diabetic
30 min postprandial (random check, 180 patients)	≥11.1	1	12.9	Diabetic

**Table 4:** Classification of patients with diabetes based on blood glucose levels and measurement timing relative to food intake.

Measurement	Defined Glucose Range (mmol/L)	Number of Patients	Observed Range	Category
Fasting (80 patients)	<4.4	1	4.2	Hypoglycemia
	4.4–7.2	42	4.9–7.2	Well-controlled
	>7.2	37	7.3–17	Not controlled
2 h postprandial (116 patients)	<4.4	2	3.3–4.3	Hypoglycemia
	4.4–10	68	4.9–9.9	Well-controlled
	>10	46	10.1–33.33	Not controlled
30 min postprandial (62 patients)	≤10	37	5.1–9.9	Well-controlled
	>10	25	10.2–21.6	Not controlled

## Data Availability

The data presented in this study are available on request from the corresponding author. The data are not publicly available due to privacy restrictions.
